# Multivalent Fc**γ**-receptor engagement by a hexameric Fc-fusion protein triggers Fc**γ**-receptor internalisation and modulation of Fc**γ**-receptor functions

**DOI:** 10.1038/s41598-017-17255-8

**Published:** 2017-12-06

**Authors:** O. S. Qureshi, T. F. Rowley, F. Junker, S. J. Peters, S. Crilly, J. Compson, A. Eddleston, H. Björkelund, K. Greenslade, M. Parkinson, N. L. Davies, R. Griffin, T. L. Pither, K. Cain, L. Christodoulou, L. Staelens, E. Ward, J. Tibbitts, A. Kiessling, B. Smith, F. R. Brennan, M. Malmqvist, F. Fallah-Arani, D. P. Humphreys

**Affiliations:** 1 UCB Pharma, 216 Bath Road, Slough, SL1 3WE UK; 2 Ridgeview Diagnostics AB, Uppsala Science Park, SE75183 Uppsala, Sweden; 3 UCB Biopharma Sprl., Chemin du Foriest, Braine l’Alleud, Belgium

## Abstract

Engagement of Fcγ-receptors triggers a range of downstream signalling events resulting in a diverse array of immune functions. As a result, blockade of Fc-mediated function is an important strategy for the control of several autoimmune and inflammatory conditions. We have generated a hexameric-Fc fusion protein (hexameric-Fc) and tested the consequences of multi-valent Fcγ-receptor engagement in *in vitro* and *in vivo* systems. *In vitro* engagement of hexameric-Fc with FcγRs showed complex binding interactions that altered with receptor density and triggered the internalisation and degradation of Fcγ-receptors. This caused a disruption of Fc-binding and phagocytosis. *In vivo*, in a mouse ITP model we observed a short half-life of hexameric-Fc but were nevertheless able to observe inhibition of platelet phagocytosis several days after hexameric-Fc dosing. In cynomolgus monkeys, we again observed a short half-life, but were able to demonstrate effective FcγR blockade. These findings demonstrate the ability of multi-valent Fc-based therapeutics to interfere with FcγR function and a potential mechanism through which they could have a sustained effect; the internalisation and degradation of FcγRs.

## Introduction

The Fcγ-receptors (FcγRs) consist of a family of IgG-binding transmembrane proteins, each with varying affinities for the IgG Fc region, isotype specificity, cell-type expression pattern and down-stream signalling pathways^1,2^. The human receptors consist of the activating receptors FcγRI (CD64), FcγRIIa (CD32a), FcγRIIc (CD32c), FcγIIIa/b (CD16a/b) and a single inhibitory receptor FcγIIb (CD32b). FcγRI has a high affinity for monomeric IgG with the other receptors having low affinity interactions^3,4^. The complexity of these receptor interactions is increased further by receptor preferences for IgG isotypes 1–4^3^. This diversity of Fcγ receptors in turn contributes to the range of functions of these receptors in immune cells such as B cells, macrophages, dendritic cells and NK cells where they can evoke a range of cellular responses including phagocytosis, cytokine release, antibody-dependent cellular cytotoxicity and antigen-presentation^5–7^. As a result, these receptors play a critical role in immunity to pathogens and tumours.

FcγRs have been directly linked to a number of diseases such as immune thrombocytopenia purpura (ITP), through FcγR-mediated platelet phagocytosis by splenic and liver macrophages, and myasthenia gravis, potentially through the destruction of AChR-expressing cells by antibody-dependent cell-mediated cytotoxicity^5^. In inflammatory neuropathies such as Guillain-Barre Syndrome (GBS) and chronic inflammatory demyelinating polyneuropathy (CIDP), macrophage Fc receptors may contribute to demyelination^8^. More generally, FcγR polymorphisms have been linked to autoimmune diseases such as systemic lupus erythematosus (SLE) and rheumatoid arthritis^9^. As a result, FcγR blockade using IVIg is a therapeutic strategy. The mechanism through which IVIg exerts its therapeutic effects is currently an area of considerable attention as efforts are made to develop more efficacious, cost-effective and safer alternatives^10,11^. A potentially important mechanism is Fcγ receptor blockade through the Fc region. In a human ITP trial using IV infusions of Fcγ fragments, the Fc fragment but not the F(ab’)_2_ was effective in treating ITP^12^. In patients undergoing IVIg treatment reduced expression of FcγRIIa on circulating myeloid dendritic cells was observed^13^ and the aggregated or cross-linked fractions of IgG appear more potent in mouse models of ITP^14,15^. We and others have therefore postulated that multivalent Fc constructs may have the potential for the treatment of immune conditions involving pathogenic antibodies^11,16–18^ (Rowley *et al*. In preparation).

The sequence and functional overlap between human and mouse receptors is incomplete and complex. While generic lessons can be learnt from studies using murine cells and tissues, the added complexity of differential engagement of human or mouse Fcs on mouse, primate or human receptors, leads to a preference for studies using human systems or mice transgenic for human receptors in order to translate Fc-based entities into effective therapeutics^19^. We generated a hexameric Fc-fusion protein with the aim of generating a uniform therapeutic agent capable of studying the interference of Fc receptors in a range of disease settings. We engineered human IgG1 and IgG4 Fc domains into hexameric forms (hexameric-Fc) by fusion of the human IgM ‘tail-piece’ to the Fc C-terminus (Supplementary Figure 1)^18,20–22^. We have investigated the consequences of Fc-receptor engagement with this protein in a range of Fc-mediated functions. Our findings highlight how the multi-valent engagement of Fc-receptors can lead to functional down-modulation of Fc receptors and disruption of Fc receptor functions *in vitro* and *in vivo*. These findings inform our understanding of the likely therapeutic benefits of multi-valent Fc-fusion proteins in clinical settings as well as suggesting mechanisms through which pathogenic multi-valent Fc complexes could disrupt the immune system.

## Results

### Binding of a hexameric Fc-fusion protein to Fc-receptors

**Figure 1:**
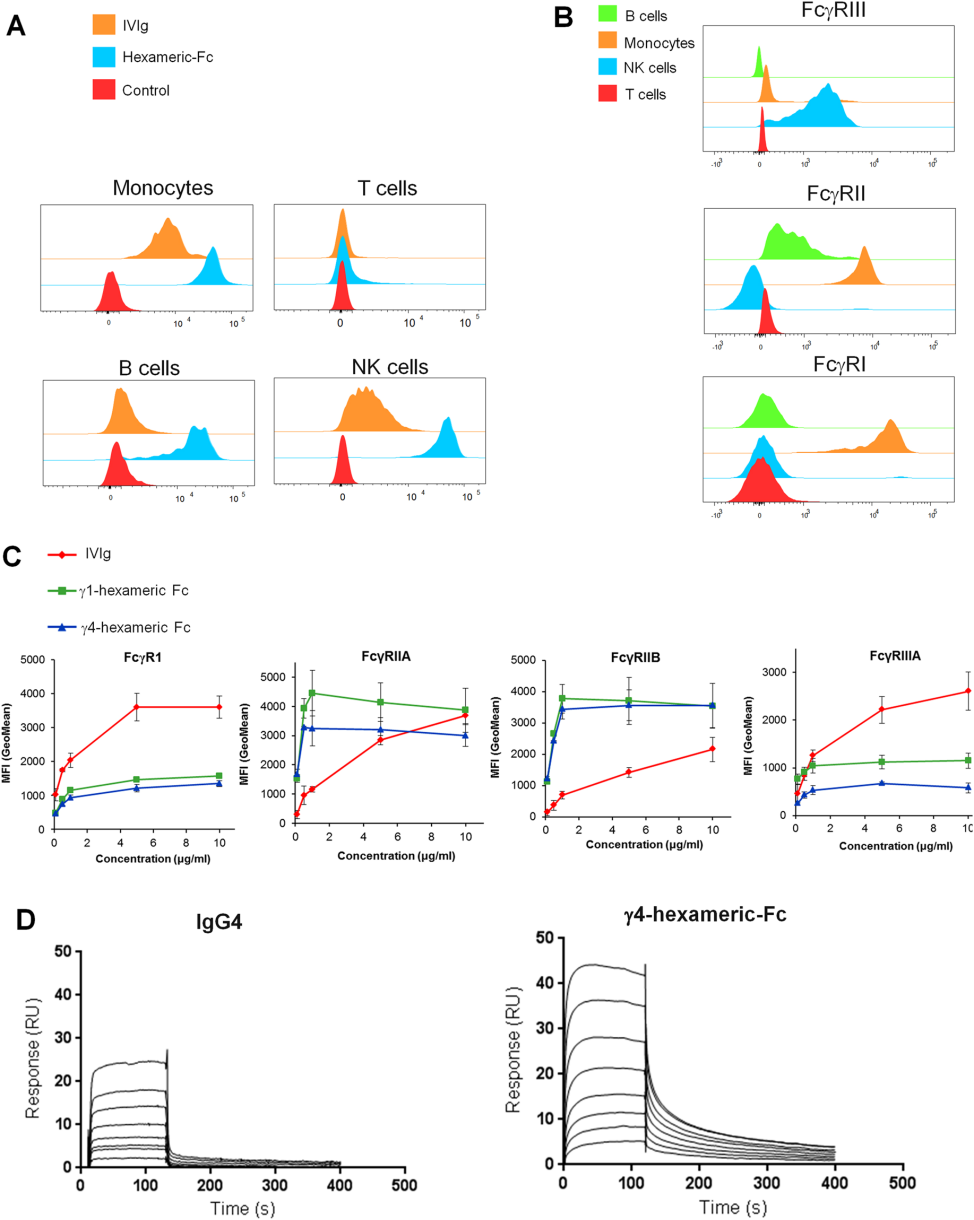
Characterisation of hexameric-Fc binding. (**A**) The binding of fluorescently tagged γ1 hexameric-Fc and IVIg to PBMC subsets was analysed by flow cytometry. Shown is 1 representative donor out of 3 independent donors. (**B**) Flow cytometric analysis of FcγR expression on PBMCs. Cells from the same donor as in (**A**) were stained with FcγR antibodies to analyse FcγRI, FcγRII and FcγRIII expression on PBMC subsets. (**C**) Binding of hexameric-Fc to HEK293 cells transfected with indicated FcγR constructs. Data shows the mean of 3 independent experiments + /- SEM. (**D**) Representative SPR traces showing the binding of γ4-hexameric-Fc and IgG4 to FcγRIIa. Hexameric-Fc was titrated in a two-fold dilution series from 1μM to 7.8 nM. IgG4 was titrated in a two-fold dilution series between 50μM and 0.39 μM.

We initially performed a characterisation of the binding of a hexameric-Fc (See methods, S. Figure 1A) and the predominantly (>95%) monomeric IVIg to peripheral blood mononuclear cells (PBMCs). As expected, cells expressing human Fcγ receptors I (CD64), II (CD32) or III (CD16), such as monocytes, B cells and NK cells showed labelling with a fluorescently-conjugated hexameric-Fc (Fig. 1A & B). T cells, which did not show expression of these Fc receptors, did not show significant labelling. To investigate more specifically the binding of multivalent ligands to FcγRs, we incubated HEK cells transfected with human FcγRI, FcγRIIA, FcγRIIB and FcγRIIIA, with IgG1 hexameric-Fc fusion protein (γ1-hexameric-Fc), IgG4 hexameric-Fc (γ4-hexameric-Fc) or IVIg. We observed dose-dependent binding of both hexameric-Fcs to all of the Fc-receptors tested (Fig. 1C). For all receptors, hexameric-Fc reached 50% of the maximal signal (Bmax) at a lower concentration than IVIg which is indicative of stronger affinity (lower KD) or avidity effects. The maximal binding however is lower for the hexameric-Fc than for IVIg on FcγRI and FcγRIIIA. Whilst this is surprising, this may be caused by the topology or steric differences between the larger hexameric-Fc and the predominantly monomeric IVIg and their modes of interaction with FcγRI and FcγRIIIA. To study the hexameric-Fc binding in more detail, we measured the binding of hexameric-Fcs by surface plasmon resonance (SPR). In this case, monomeric IgG showed less stable interaction and a more rapid return to baseline in the dissociation phase ‘off-rate’ than hexameric-Fc (Fig. 1D). However, the binding of hexameric-Fc to the Fcγ-receptors did not show a single on and off rate which suggested additional complexity in the binding interaction.

**Figure 2:**
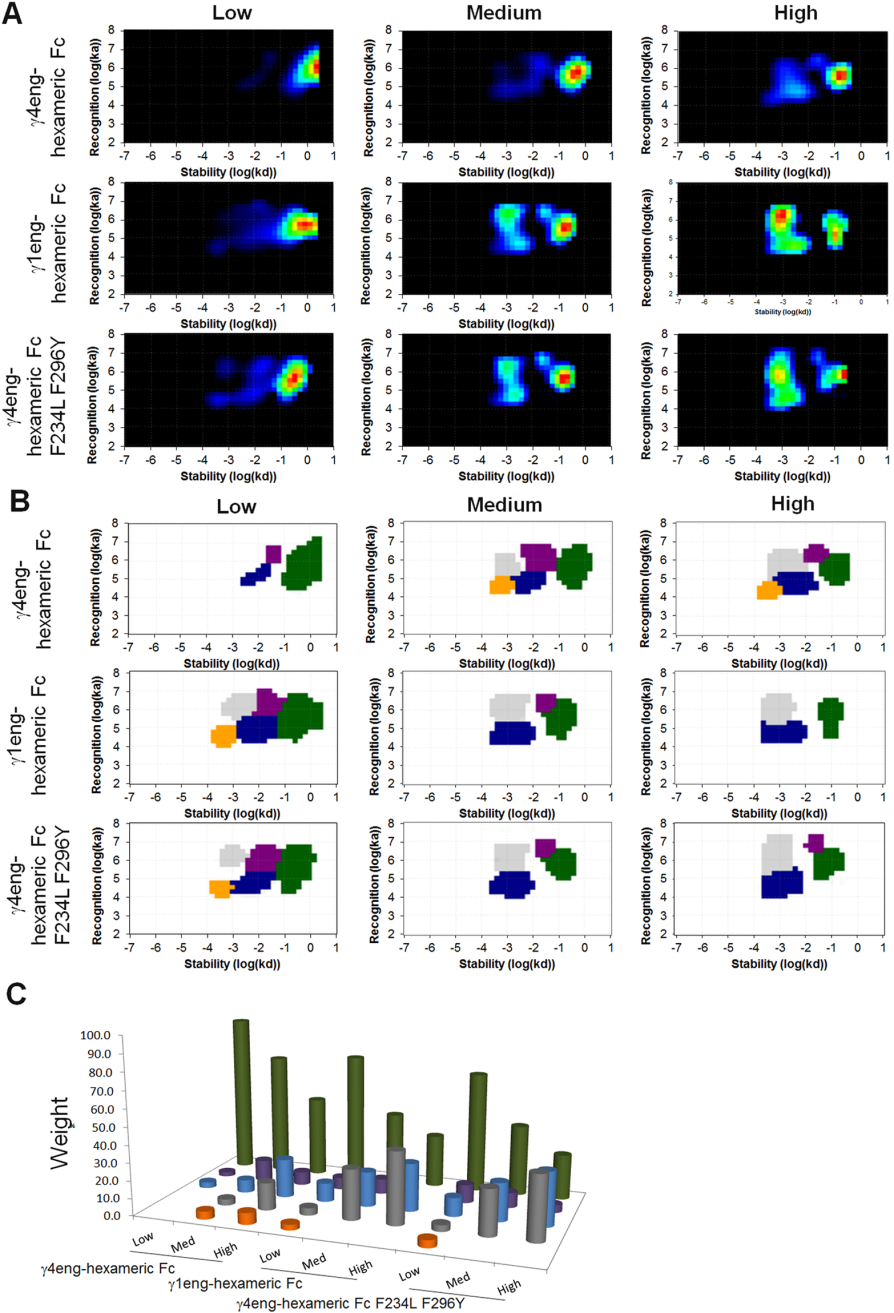
SPR and Interaction Map analysis of hexameric-Fc binding to FcγRIIIa. (**A**) Interaction Map of the SPR binding traces of γ4eng, γ1eng and γ4eng hexameric-Fcs to different surface concentrations of immobilised recombinant FcγRIIIa, as analysed by BIAcore at a range of concentrations between 7.8 and 100 nM. The immobilisation level was 10 pg of protein per square mm (response units, 10RU) (low), 32 RU (medium) and 85 RU (high) for the different experiments. Each peak corresponds to a contributing interaction process. Red shows strongly contributing interactions whilst blue shows weaker contributions. (**B**) Definition of peaks in TraceDrawer to obtain information about k_a_, k_d_, K_D_ and weight of each peak. (**C**) The weight of the different peaks in each experiment.

Having observed multiple off- and on-rates as well as potential additional modes of binding interaction, we performed SPR binding studies at a range of concentrations of three sequence variants of hexameric-Fcs to recombinant FcγIIIa immobilised on a chip at three different surface densities. The hexameric-Fcs tested were an IgG4 isotype hexameric-Fc engineered to contain the CH3 domain of IgG1 as this improved large scale production properties (γ4eng-hexameric-Fc), an IgG1 isotype hexameric Fc containing L234F P331S mutations to reduce platelet and complement activation (γ1eng-hexameric-Fc), and an IgG4 isotype hexameric-Fc which had F234L F296Y mutations introduced to increase blockade of FcγR mediated phagocytosis (γ4eng-F234L F296Y hexameric-Fc) (Rowley *et al.* In preparation). Data was analysed using the interaction Map® method which allows the analysis of heterogeneous molecular interactions from real-time binding curves^23,24^. The Interaction Map generated a two-dimensional distribution of k_a_ and k_d_ with the colour giving a measure of how much a particular interaction contributes to the binding (Fig. 2A). The heat maps show the heterogeneity of the binding with a number of interaction processes. Peaks were defined as shown in Fig. 2B to calculate the weight of each peak. Figure 2C then shows the distribution of peaks and their weight for each experiment. The interaction at the low target surface density was relatively homogeneous, with a major contributing interaction corresponding to approximately 90% for γ4-eng hexameric-Fc and 70% for γ1-eng hexameric-Fc and γ4-eng F234L F296Y hexameric-Fc (Fig. 2C). At higher densities the interaction became more heterogeneous and the contribution of the major interaction was reduced, in particular for γ1eng hexameric-Fc and γ4eng hexameric-Fc. Instead, the contribution of higher affinity peaks (primarily blue and silver) increased. The density dependency of the heterogeneity suggests avidity effects, i.e. a more multivalent binding was possible if the targets were close enough.

The peaks appeared at similar positions for all three hexameric-Fcs. The affinity was higher for all additional peaks (1–44 nM) than the major green peak (420 nM) which is in line with the hypothesis that the green peak corresponds to a monovalent binding and the other peaks are the result of a multivalent binding. It is unclear if the avidity effects can be simplified into “one peak for each binding arm”, or if they are more complex with e.g. synergistic effects.

Taken together, these results show that in multivalent Fc-containing proteins show multiple binding interactions that are not necessarily predictable *a priori*.

### Internalisation and degradation of Fcγ-receptors following hexameric Fc-engagement

**Figure 3:**
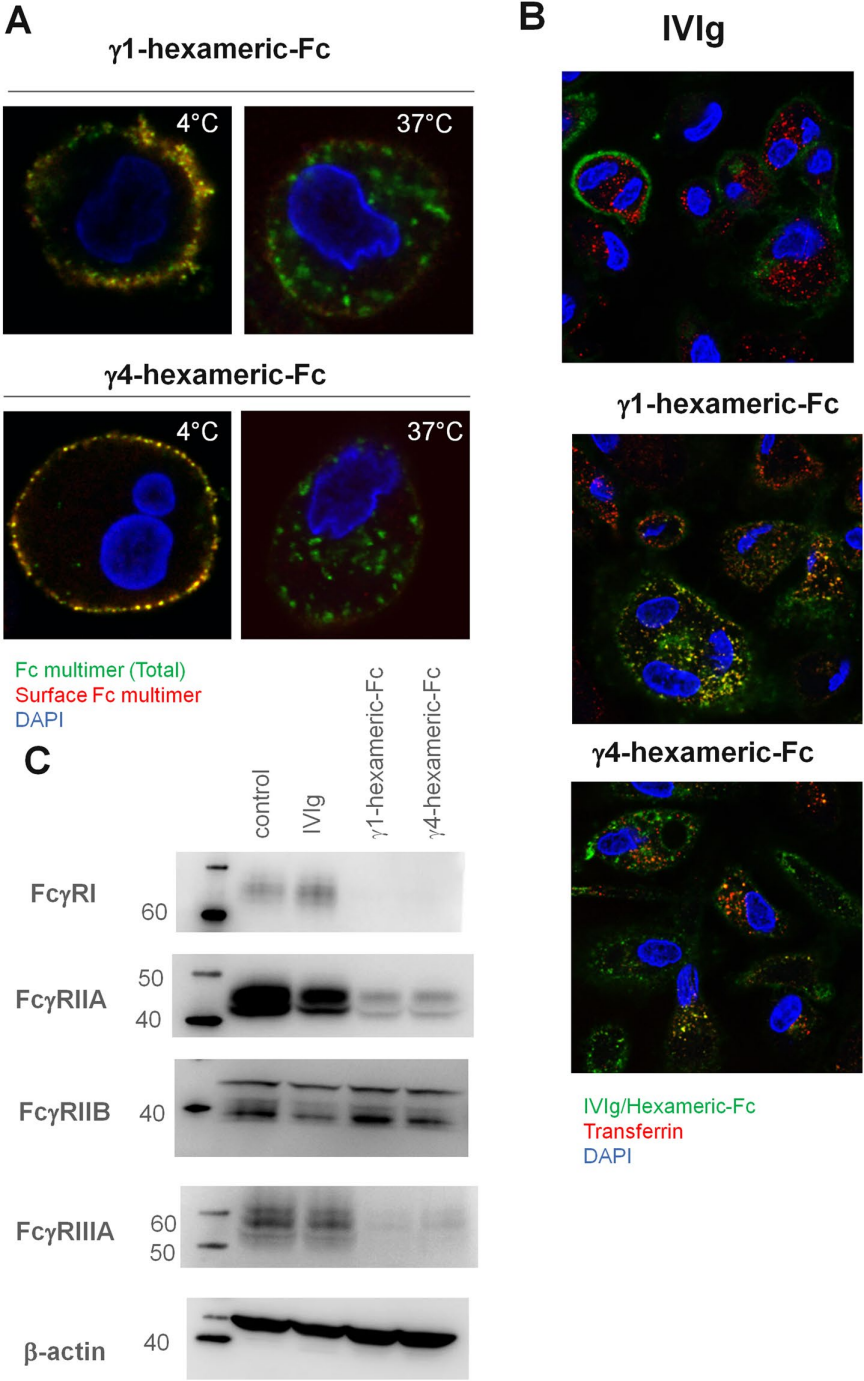
Internalisation of hexameric-Fc and degradation of Fcγ-receptors. (**A**) Macrophages were incubated with AF488-conjugated hexameric-Fc at either 4 °C or 37 °C for 30 minutes, followed by incubation with anti-human AF647 at 4 °C to label hexameric-Fc remaining at the surface. Cells were then fixed and imaged by confocal microscopy. Representative images taken from one of three independent experiments is shown. (**B**) Macrophages incubated at 37 °C with AF488-conjugated hexameric-Fc or IVIg in the presence of AF568-conjugated transferrin. Cells were fixed and imaged by confocal microscopy. (**C**) Macrophages were incubated with hexameric-Fc or IVIg for 24 hours. Cell lysates were analysed by Western blotting using antibodies against the Fc-receptors cytoplasmic domains. One representative blot from three independent experiments is shown.

The engagement of Fcγ-receptors by multivalent ligands has been associated with their internalisation^25^. To test the effect of hexameric-Fc on FcγRs, we differentiated human macrophages from peripheral blood monocytes. We then incubated macrophages with a fluorescently-labelled hexameric-Fc either at 4 °C or 37 °C for 30 minutes. The cells were then cooled to 4 °C and any hexameric-Fc that had remained at the cell surface was detected with a fluorescently-conjugated secondary antibody against human IgG. The cells incubated at 37 °C showed a considerable number of intracellular vesicles containing hexameric-Fc that were not labelled by the secondary antibody and little detectable surface hexameric-Fc labelling, suggesting the hexameric-Fc was efficiently internalised (Fig. 3A). In contrast, the macrophages labelled at 4 °C showed plasma membrane staining with considerable co-localisation of the two fluorophores, indicating the hexameric Fc had remained at the cell surface at this temperature (Fig. 3A). To further investigate this process, macrophages were co-incubated with fluorescent hexameric-Fc or IVIg and fluorescent-transferrin (to mark the clathrin-mediated endocytic pathway) and incubated at 37 °C (Fig. 3B). We observed significant co-localisation of the hexameric-Fc with transferrin but limited co-localisation with IVIg indicating the efficient internalisation of a hexameric-Fc ligand by these cells. The co-localisation with transferrin also suggested the transport of the hexameric-Fc into the recycling endosome.

To test the fate of the FcγRs in this system, we performed Western-blotting for the receptors after exposure to IVIg or hexameric-Fc for 24 hours at 37 °C. We observed significant degradation of the stimulatory FcγRs: FcγRI, FcγRIIA and FcγRIIIA after contact with hexameric-Fc (Fig. 3C). Interestingly, we did not observe degradation of the inhibitory FcγRIIB which is consistent with reports in transfected systems of this receptor recycling in response to immune complex engagement^26,27^. In contrast, following incubation with IVIg, we did not observe significant degradation of Fc receptors. In summary, these results confirm the rapid internalisation of hexameric-Fc and the subsequent degradation of activating but not inhibitory FcγR, a mechanism absent with IVIg.

### Disruption of *in vitro* Fc-receptor functions following incubation with hexameric-Fc

**Figure 4:**
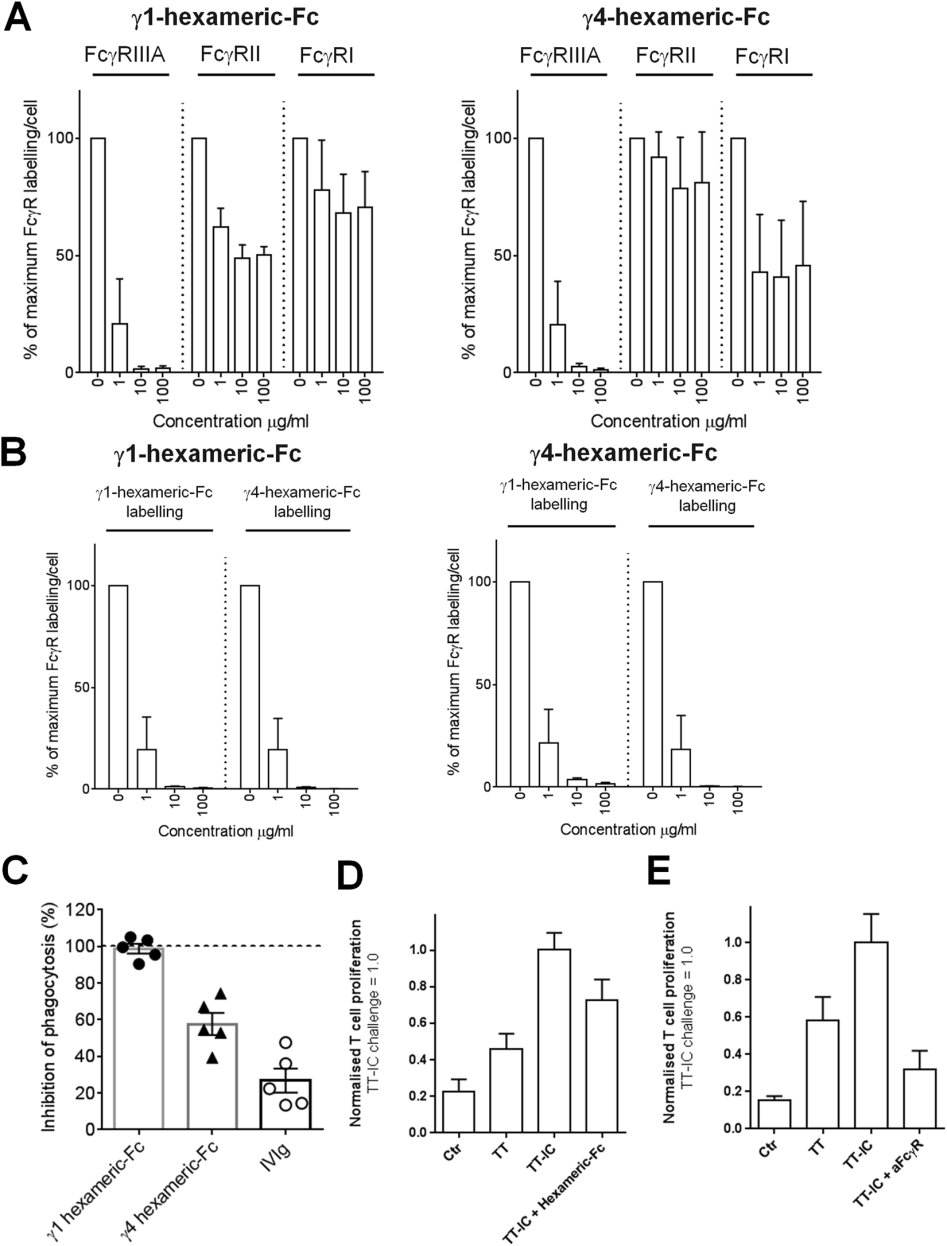
Incubation with hexameric-Fc interferes with Fcγ receptor mediated function Macrophages were incubated with hexameric-Fc for 2 hours. FcγRs were then labelled at 4 °C using fluorescently-conjugated antibodies (**A**) or Fc-binding capacity (**B**) assessed using fluorescently conjugated hexameric-Fc. Cells were then fixed, DAPI-labelled and fluorescence quantified using by automated-fluorescence microscopy. (**C**) Hexameric-Fc inhibits macrophage phagocytosis. Human monocyte-derived macrophages were co-cultured with autologous CFSE-labelled B cell targets in the presence of 0.1 μg/ml anti-CD20 to opsonise. IgG1 or IgG4 wild type hexameric-Fc or IVIg were added at 100 µg/ml. The disappearance of target cells was measured by flow cytometry after 18hrs and plotted as % inhibition of total antibody-dependent phagocytosis. Data are the mean of 5 individual donor experiments ± SEM. (**D & E**) T cell (CD3^+^) proliferation after tetanus toxoid (TT) immune complex (TT-IC) challenge. CellTrace Violet labelled PBMCs were incubated with TT (1 µg/ml) or pre-formed TT-ICs (to a total of 1 µg/ml of TT) for 6d. During this period, cells were co-incubated with either γ1 hexameric-Fc (50µg/ml, **D**) or antibodies against FcγRIIA/B and FcγRIIIA/B (20 µg/ml, **E**). Proliferation was assessed by CellTrace Violet dye dilution in CD3^+^ T cells, and normalised and pooled data is expressed as mean ± SEM. T cell responses of 5 individual donors from at least 2 independent experiments per condition.

The high-affinity binding of multi-valent immune complexes and the resulting Fc-receptor blockade/degradation could disrupt the function of FcγRs. This could present a therapeutic modality to block FcγRs in autoimmune or inflammatory settings^11,21^ and also explain the immune-complex-mediated FcγR disruption observed in chronic viral infection^28,29^. We incubated human monocyte-derived macrophages with hexameric-Fc for 2 hours and observed a reduction in the surface labelling of FcγRs. FcγRIII (CD16) was especially effected by both IgG1 and IgG4 hexameric-Fc and to a lesser extent FcγRIIA (CD32a) after exposure to IgG1 hexameric-Fc (Fig. 4A). The ability of cells to bind fluorescent hexameric-Fc as model FcγR-ligands was almost completely abolished by ≥1μg/ml of both γ1 and γ4 hexameric-Fc illustrating the global potency of receptor blockade by hexameric-Fc (Fig. 4B). We then proceeded to test further FcγR functions. Initially, we performed a flow-cytometry-based phagocytosis assay (Fig. 4C). Human macrophages were incubated with autologous B cell targets that had been coated with anti-CD20 IgG1 monoclonal antibody to trigger Fc-mediated phagocytosis. In the absence of anti-CD20 mAb we observed almost no phagocytosis of B cells, indicating that this assay captured predominantly Fc-dependent phagocytosis (data not shown). Co- incubation of macrophages with hexameric-Fc showed inhibition of phagocytosis, with a γ1-based hexameric-Fc acting more potently (99% inhibition) than a γ4-based hexameric-Fc (58% inhibition). However, both isotypes of hexameric-Fc were more potent at inhibition of phagocytosis than IVIg (28% inhibition).

Fc receptors play an important role in antigen presentation through the uptake of immune complexes (ICs) in antigen-presenting cells^30^. Having observed the sparing of FcγRIIB in Fig. 3C and previous observations of the role for this receptor in antigen presentation^31^, we tested whether or not hexameric-Fc was capable of blocking this Fc-mediated function. We focused in particular on γ1-hexameric-Fc as a model protein as it showed the greatest potency in blocking phagocytosis (Fig. 4C). In this system, generation of tetanus toxoid ICs (TT-IC) was associated with an increase in T cell proliferation when compared to uncoated tetanus toxoid (Fig. 4D and E). In this system however, the γ1 hexameric-Fc could not inhibit TT-IC-mediated T cell proliferation (Fig. 4D). This is in contrast to a cocktail of Fcγ-receptor blocking antibodies which effectively reduced proliferation to baseline levels (Fig. 4E). Taken together, this highlights that while hexameric-Fc can be an effective blocker of some FcγR-mediated functions, other functions may be spared.

### Prolonged disruption of Fcγ-receptor functions *in vitro* and *in vivo* with hexameric Fc

**Figure 5:**
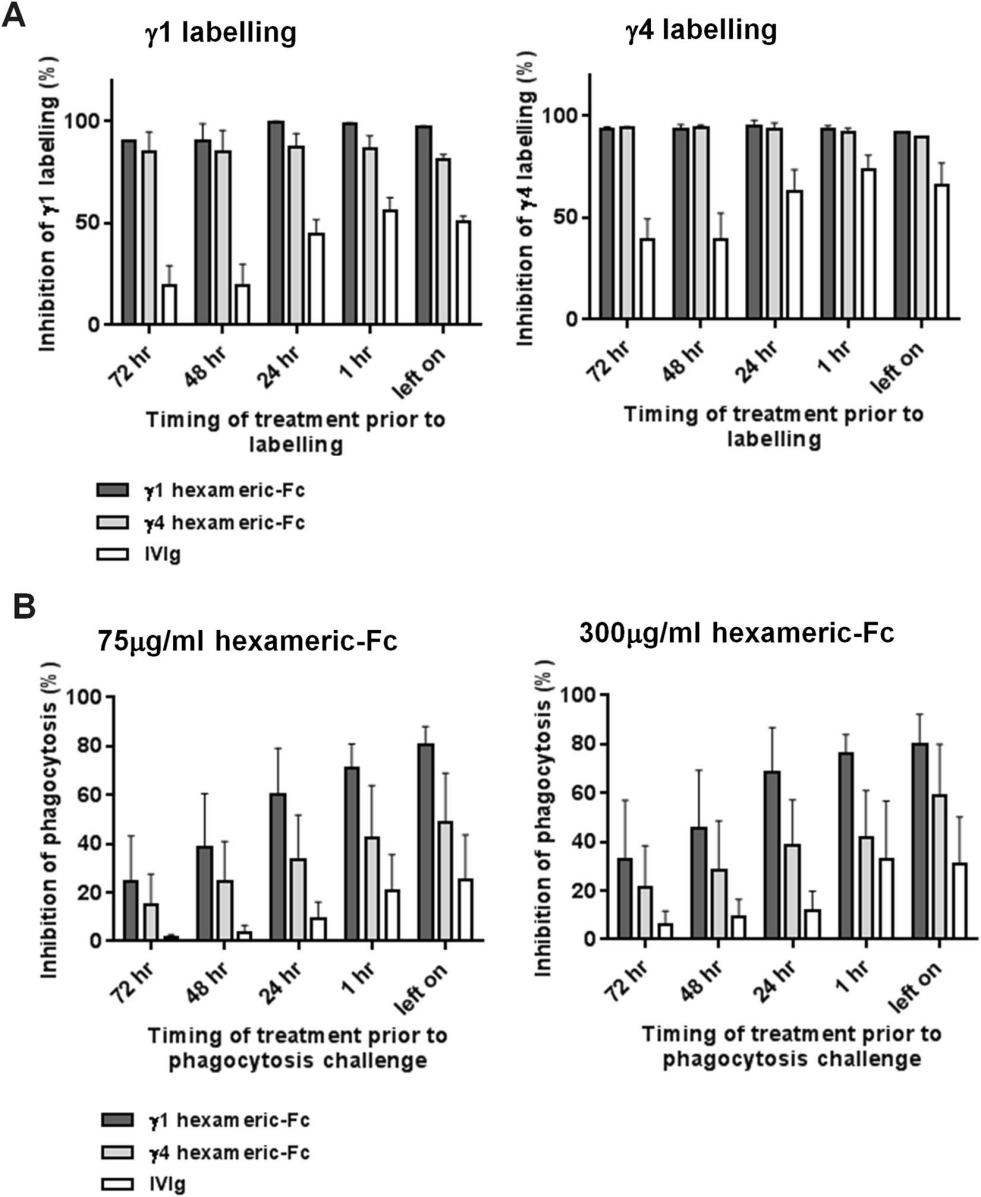
*In vitro* disruption of FcγR function. (**A**) Macrophages were incubated with hexameric-Fc s or IvIg at 75 µg/ml for 1 hour. Cells were then washed and incubated for the indicated period. Cells were then labelled with fluorescently-conjugated hexameric-Fc. Data shows means from three donors ± SEM. (**B**) Pre-incubation with hexameric-Fc inhibits phagocytosis for 72hrs. Human monocyte-derived macrophages were incubated with the indicated hexameric-Fcs or IVIg at 75 µg/ml or 300 µg/ml for 1 hour, washed and cultured for 1 to 72 hours before co-culture with autologous CFSE-labelled B cell targets in the presence of 0.1 μg/ml anti-CD20. The disappearance of target cells was measured by flow cytometry after 18hrs and plotted as % inhibition of total antibody-dependent phagocytosis. Data are the mean ± SEM for three donors.

Having observed that hexameric-Fc could cause degradation of FcγRs we wished to test whether this caused a disruption of function. This effect has potentially important implications for the use of multi-valent Fc-based therapeutics and could also contribute to immune dysfunction in diseases involving chronic generation of immune complexes^28,29^. Macrophages were incubated with hexameric-Fc, washed and then incubated for a recovery period in which hexameric-Fc was absent. We observed a reduction in the ability of the cells to bind Fc-containing ligands which was maintained for up to 72 hours, as judged by fluorescent hexameric-Fc labelling (Fig. 5A). Furthermore, after 72 hours, Fc-mediated phagocytosis of anti-CD20 coated B cells was still impaired following an initial 1 hour incubation with γ1eng-hexameric-Fc or γ4eng-hexameric (Fig. 5B). In contrast, IVIg did not show inhibition of Fc-mediated function at these time-points. Although at the extended time-points we did observe a reduction in cell counts (25% after 24 hours, 50% after 48 hours, 65% after 72 hrs of initial cell counts, data not shown), there was no difference in reduction between those treated with IVIg (where FcγR function recovered) and those treated with hexameric-Fc. This suggested that in these *in vitro* experiments, the rate of FcγR re-expression was not sufficient to fully restore the Fc-binding capacity or full phagocytic function.

Nevertheless, we did observe a difference between the duration of reduction in Fc-binding and phagocytosis (Fig. 5A vs B), with phagocytosis activity appearing to return more rapidly than the expression of the majority of the FcγRs. To try to understand this effect further, we performed surface labelling of receptors on macrophages that had been treated as described above (S. Figure 2). FcγRIIA and FcγIII showed an almost complete and sustained reduction in labelling. Although we do not have an antibody that specifically recognises FcγRIIB, comparing labelling of the pan-FcγRII clone FLI8.28, and the more FcγRIIA-selective IV.3^32^, revealed a basal labelling that was not disrupted by hexameric-Fc incubation. This suggested that FcγRIIB was not disrupted (consistent with its lack of degradation, Fig. 3C) and could perhaps contribute to phagocytosis. In addition, although labelling was close to base-line levels and variable, FcγRI also appeared to show surface labelling and could potentially contribute to phagocytosis. In summary, these data show hexameric-Fc engagement of FcγRs results in a disruption of Fc-mediated function *in vitro* that did not appear to recover for a prolonged period.

**Figure 6:**
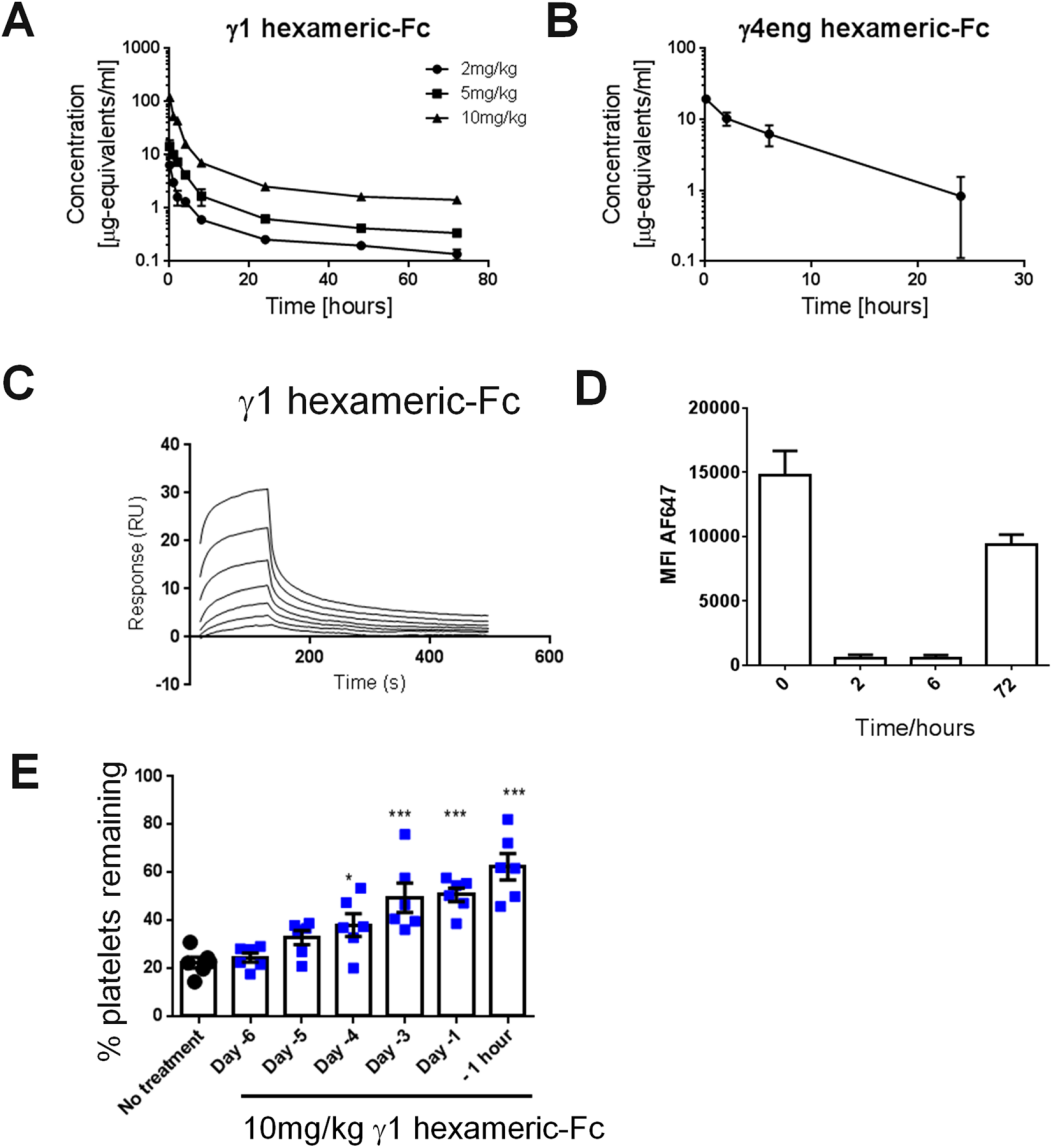
*In vivo* effects of hexameric-Fc. (**A**) ^125^I γ1 Hexameric-Fc was administered to mice at 0.5 mg/kg, 2 mg/kg or 10 mg/kg. At indicated timepoints, plasma was collected from three mice per timepoint and concentration of protein bound radioactivity in plasma determined by direct measurement in a gamma counter. Values were corrected to calculate μg-equivalents of hexameric-Fc per mL of plasma. (**B**) γ4eng F234L F296Y hexameric-Fc administered to cynomolgus monkeys by IV bolus at 1 dose of 2 mg/kg. Concentrations of hexameric-Fc and smaller and larger human Fc-containing moieties were detected in plasma by mass spectroscopy. n of 3 animals, ± SEM. (**C**) Binding of γ1-hexameric-Fc to immobilised recombinant FcRn was investigated by SPR. Hexameric-Fc was titrated in a two-fold dilution series from 2.5μM to 39 nM. (**D**) To assess hexameric-Fc mediated FcγR blockade in cynomolgus monkeys, whole blood samples were collected after a 30 mg/kg IV dose of γ4eng F234L F296Y hexameric-Fc. Surface labelling of samples was carried out to identify monocytes (CD14^+^) and occupancy of FcγRs assessed using a AF647-conjugated γ4eng F234L F296Y prior to analysis by flow-cytometry. 3 animals, ± SEM. (**E**) To assess the effect of hexameric-Fc in an ITP model, 10 mg/kg hexameric-Fc was administered to mice IV, at the timepoints indicated, prior to addition of anti-CD41 (MWReg30) to induce platelet depletion. Whole blood samples were taken immediately prior to and 24 hour post anti-CD41 in order to determine platelet numbers. n = 6 mice per group, graph shows mean ± SEM, *=p < 0.05, ***=p < 0.01, by ANOVA and Dunnetts multiple comparison test.

Whilst hexameric-Fc could cause a disruption of *in vitro* FcγR function we wished to test whether this effect was mimicked *in vivo*. Following IV administration of a ^125^I-labelled dose of γ1-hexameric Fc in mice, concentrations of Fc in plasma decreased rapidly and had reduced approximately 50-fold within 24 hours of dosing (Fig. 6A). By this time point, at the 10 mg/kg dose, monomeric Fc accounted for most of the remaining radioactivity with the hexameric fraction at a concentration of approximately 0.006μg-equivalents/ml.

There are significant species differences in FcγRs^33^ so we therefore performed additional experiments in cynomolgus monkeys to help confirm the translational relevance of these findings. Dosing of the γ4eng F234L F296Y-hexameric-Fc led to a similar rapid clearance with hexameric-Fc below the limit of detection after 48 and 72 hours (Fig. 6B). In both cases, the hexameric-Fcs were cleared substantially faster than that observed for an IgG in mice or cynomolgus monkeys^34^. The cause of the rapid clearance of the hexamers is likely related to binding to Fcγ-receptors. While it is possible that hexameric-Fc could interfere with binding to the neonatal Fc receptor (FcRn), the receptor responsible for recycling of IgG via pH dependent binding to the Fc^35–37^; hexameric-Fc still showed binding to FcRn receptors by SPR (Fig. 6C) and mutations that increased or ablated binding to FcRn did not substantially alter either hexameric-Fc PK or clearance of IgG in mice (S. Figure 3). Taken together, the rapid clearance appears more consistent with hexameric-Fc binding to FcγRs on the cell surface with subsequent elimination by internalisation and degradation. These data are consistent with studies on the saturation of the FcγR dependent reticulo-endothelial systems of animals, where 200–250 mg/kg of antibody immune complexes were required to approach receptor saturation^38,39^. Hence the 10 mg/kg dose used here was unlikely to approach these target saturation limits. At the 10 mg/kg dose in mice, we did observe transient signs of piloerection, hunched posture and decreased activity, all of which resolved within 30 minutes and which were not observed in cynomolgus monkeys at the 30 mg/kg dose of the γ4eng F234L F296Y-hexameric Fc. We hypothesised that this could have been caused by cytokine release as a result of FcγR cross-linking. While we did observe transient elevation of Il-6, Il-10 and KC (S. Figure 4) we were unable to detect IFNγ, TNFα or Il-1β.

To understand whether treatment with hexameric-Fc disrupted Fc-receptor function *in vivo*, we measured the Fc-binding capacity of cells *ex vivo* whole-blood using a fluorescently-conjugated hexameric-Fc. We then measured the labelling of monocytes (as exemplar phagocytic cells) with fluorescent hexameric-Fc in whole blood samples from cynomolgus monkeys that had been previously dosed intravenously with 30 mg/kg γ4eng F234L F296Y-hexameric Fc. The Fc-binding capacity of monocytes was reduced at the 2 and 6 hour time-points and although we were unable to sample at 24 h or 48 h timepoints, still appeared slightly reduced after 72 hours suggesting that a hexameric-Fc was capable of disrupting FcγR function in a species with translational relevance to man (Fig. 6D).

Immune thrombocytic purpura (ITP) in humans is characterised by the presence of IgG1/3 and IgM antibodies^40^ against platelet surface receptors resulting in their clearance via the reticuloendothelial system in the liver and spleen^41^. *En masse* blockade of or destruction of FcγR-bearing phagocytic cells has been shown to reduce platelet destruction^42^. To test if disruption of FcγR function by hexameric-Fc can have therapeutic relevance for ITP we used an established mouse model of ITP that involves bolus administration of an anti-platelet IgG which targets platelets for rapid phagocytic destruction^43^ in a similar way as observed in humans, leading to a drop in circulating platelet numbers.

In this ITP model, we tested the duration of FcγR blockade by dosing mice with hexameric-Fc at a range of time-points before administration of the anti-platelet IgG. Platelet numbers were maintained even at time-points where based on mouse PK data, we would have expected a significant reduction of drug in circulation (Fig. 6E). Platelets are substantially protected from phagocytic elimination up to 3 days after bolus hexameric-Fc, even though the hexamer is ~90% cleared from the circulation in 24 h. Platelet protection is still statistically significant 4 days after a single bolus dose of hexameric-Fc. These findings suggest that FcγR function can be disrupted *in vivo* following exposure to hexameric-Fc.

## Discussion

We have constructed hexameric-Fc with the aim of generating a potent, recombinant molecule capable of blocking FcγR-mediated functions. Hexameric-Fc showed binding to all FcγRs tested and showed evidence of multiple binding interactions dependent on receptor density. Despite some variation in maximal binding to the FcγRs, the interaction nevertheless was sufficient to reduce surface labelling of FcγRIIIA and FcγRII. Additionally, this interaction led to the blockade of Fc-binding and phagocytosis. Furthermore, consistent with other reports, the interaction of FcγRs with hexameric-Fc at 37 °C led to internalisation and degradation of the activating FcγRs *in vitro*. The inhibitory FcγRIIB appears to be spared from degradation after engagement by hexameric-Fc. If this phenomenon was also observed with other agents it may be consistent with an evolutionary mechanism to re-establish immune homeostasis following prolonged exposure to immune complexes^27,44^. This may have implications for potential clinical application of hexameric and other multivalent forms of Fc.

An additional consequence of receptor internalisation is the potential for sustained disruption of FcγR function. This becomes especially important as we also observed rapid clearance of hexameric-Fc and this rapid clearance may pose challenges for the clinical development of Fc-based therapeutics. We observed that, in contrast to IVIg, functional blockade of cells *in vitro* persisted after exposure to the hexameric-Fc. This was consistent with receptor internalisation and destruction. *In vitro* blockade of Fc-binding persisted for up to 72 hours after wash out of hexameric-Fc with human cells. Phagocytic function similarly showed a prolonged disruption *in vitro* albeit with some recovery. This may represent compensatory phagocytic mechanisms, phagocytosis sustained by a limited number of FcγRs, or the efficient phagocytosis of a highly opsonised particle.

*In vivo*, although the situation is more complex as result of cellular renewal, we observed a rapid clearance of hexameric-Fc. Despite this rapid clearance, in a mouse model of ITP, we observed a degree of blockade of phagocytic destruction of platelets for up to 4 days. Although there were limitations to the cynomolgous experiments in terms of samples we are able to collect, hexameric-Fc nevertheless showed a clear blockade of FcγR function at the 6 hour time point. This showed that multivalent Fc-based therapeutics can block FcγRs in a species with translational relevance for humans, and that they potentially may have a durable action that outlives their relatively rapid clearance from the circulation. Nevertheless, more comprehensive *in vivo* studies with matched time-points and hexameric-Fcs would be required to fully conclude a sustained inhibition.

Multivalent engagement of FcRn could be a desirable property in a therapeutic as it could enhance clearance of pathogenic IgG antibodies^45^. It could also contribute to subversion by viral immune complexes as it may interfere with FcRn-mediated antigen presentation^46^. Additionally, multivalent engagement of FcRn can lead to lysosomal trafficking^47^ and hence increased presentation of self-antigens. We tested to see whether wild-type and mutant forms of hexameric-Fc could increase the clearance of serum IgG by interference with FcRn function. Although variants were shown to have improved FcRn binding, no measurable increase in IgG clearance was observed. PK studies demonstrated the short serum half-life in mice of the hexameric-Fc perhaps consistent with a large cellular sink for target binding. Taken together, in this format at least, clearance of IgG through FcRn blockade does not seem a likely therapeutic mechanism for hexameric-Fc.

The FcγR-mediated uptake of immune complexes can lead to enhanced MHC-II-mediated antigen-presentation and cross-presentation, and is likely to play a critical role in tumour immunity^30^. In chronic viral infections however, recent reports have demonstrated that immune complexes, through FcγR engagement, can cause a sustained disruption in humoral immunity^28,29^. Surprisingly we were unable to observe an inhibition of antigen-presenting function after quantitative blockade of FcγR by hexameric-Fc. In our experiments and those of others FcγRIIB appears to be spared from degradation during engagement by multi-valent or high-affinity ligands^27,44^ potentially leaving it free to take up immune complexes with subsequent antigen presentation^31,48^. This receptor has a relatively low affinity for IgG^3^ but is effectively bound by hexameric-Fc *in vitro*. We failed to reduce immune-complex mediated antigen-presentation and subsequent T cell proliferation with hexameric-Fc, whereas a cocktail of anti-FcγR antibodies blocked immune-complex driven antigen presentation. This suggests that a high-affinity interaction may be required to affect this type of biology. This has implications for therapeutic utility for multivalent-Fc-based therapeutics as diseases involving an *en masse* blockade of a phagocytic component such as ITP may find better utility than those requiring interruption of self-antigen presentation in immune complexes.

FcγR cross-linking is a well-established mechanism of triggering cytokine release^7^. This is therefore a significant consideration in the development of FcγR targeted therapeutics as the balance between blocking FcγR function and FcγR activation is likely to determine the therapeutic benefit of these agents. We observed limited cytokine release in mice whilst no cytokine was observed in cynomolgus monkeys at the time-points tested. Given the significant species differences in receptor repertoire, function, distribution and binding affinity between mice and non-human-primates/humans^19^, this highlights the importance of human relevant systems for studying the effects of hexameric-Fc.

It has recently been demonstrated that immune complexes formed during chronic LCMV infection in mice can disrupt a range of FcγR functions^28,29^. The blockade/down-regulation of FcγRs following hexameric-Fc exposure may potentially mimic some of the effects observed with viral immune complexes. In inflammatory diseases, this effect may provide a short term clinical benefit for multivalent-Fc-based therapeutics as *en masse* blockers and down modulators of activating receptors whilst leaving the inhibitory FcγRIIB intact. Nevertheless, these effects have to be carefully balanced with the clear acute risk of pro-inflammatory responses observed upon Fc-receptor cross-linking and the chronic risks of increased infections and cancers due to long term *de facto* humoral immune suppression. Overall, we find hexameric-Fc provides an excellent research tool to study this biology in detail and offers some prospect of alternative treatments for human disorders involving phagocytic destruction of host cells and tissues.

## Methods

### Generation of hexameric-Fc and IVIg

Human IgG1 and IgG4 Fc with mature N-termini starting with an IgG1 core hinge (CPPC) were each directly fused at their C-terminal lysine residues to the 18 amino-acid C-terminal extension or ‘tail-piece’ (PTLYNVSLVMSDTAGTCY, tp) of human IgM (S. Figure 1A). The hexamer has a 100% human sequence, lacking mutations or insertions^21,49^ both important with regards to potential immunogenicity risk. The tail-piece contains a target motif for N-linked glycosylation and a penultimate cysteine known to be involved in IgM^575^ and IgA^471^ polymerisation^50,51^. Human IgG1 and IgG4 Fc-tp fused constructs (γ1-hexameric-Fc and γ4 hexameric-Fc, respectively) were expressed in a transient CHO system and we observed good levels of expression from all constructs (450 mg/L ± 150 mg/L). Protein A-purified hexamer were typically 70–80% and 30–40% hexamer for IgG1 and IgG4 constructs respectively (S. Figure 1B) and >98% hexamer for both IgG1 and IgG4 constructs on analytical HPLC after SEC (S. Figure 1C).

IVIg from Gammunex was buffer exchanged into PBS. HPLC analysis showed the product used in these experiments was comprised of 95% monomer, 5% dimer and undetectable levels of higher molecular weight species or aggregate.

### Macrophage Differentiation

Healthy volunteer samples from blood cones were obtained from donations through the National Blood Service which obtained signed consent and under the Ethics approval 10/H0606/40 reviewed by Oxfordshire C Research Ethics Committee on 12th August 2010. To prepare macrophages (HMDM), human peripheral blood mononuclear cells (PBMC) were first isolated from blood cones by density-gradient centrifugation. Monocytes were selected by incubating the PBMCs for 1.5hr at 37 °C in tissue culture flasks, followed by removal of non-adherent cells. Adherent monocytes were differentiated into macrophages by 7 day culture in 100ng/ml macrophage-colony stimulating factor (M-CSF, R&D Systems).

### Flow cytometric analysis of FcyR expression on PBMCs and multimer binding

All healthy volunteer blood samples were collected according to HTA regulations 2004. UCB is a long-term license holder, number 12504, since September 2013. All healthy volunteers signed a consent form, records of which are stored locally according to the HTA regulations. PBMCs from 3 independent donors were isolated using density gradient separation (Leukosep tubes, Greiner BioOne, see manufacturer’s instructions). For flow cytometric determination of cellular PBMC subsets, the following antibody lineage cocktail was used: CD3-PECy7 (BD Pharmingen), CD14-AlexaFluor 700 (BD Pharmingen), CD19-Pacific Blue (BD Horizon), CD56-APC-eFluor 780 (eBiosciences). In order to study FcyR expression, 2 × 10^5^ PBMCs were incubated for 30 minutes on ice with the lineage antibody cocktail and the following additional FACS antibodies: CD16-V500 (BD Biosciences), CD32-FITC (Stemcell Technologies), CD64-PE (BioLegend), HLA-DR-PerCP (BD). For reagent binding studies, multimer (Hexamer, IgG1, wt) or IVIg (Gamunex-c) was fluorescently tagged using an antibody labelling kit (Thermo Fisher, Alexa Fluor® 647 Antibody Labeling Kit) according to the manufacturer’s instructions. PBMCs were incubated for 1 hour with the lineage antibody cocktail and A-647 tagged reagents (multimer: 8μg/ml; IVIg: 25μg/ml). An antibody isotype control (mouse IgG1k-A647, BD Pharmingen) was used as a control. Stained PBMCs were then washed twice and analysed on a Fortessa flow cytometer (BD Biosciences) with subsequent data analysis using FlowJo (TreeStar).

### Flow cytometric analysis of FcγR expression on HEK

Stable cell lines expressing full length human Fc receptors I, IIA, IIB and IIIA were created in HEK 293 cells which were cultured in suspension in FreeStyle 293 Expression Medium (Life Technologies) at 37 °C, 8% CO2 and 120 rpm in the presence of 0.5 mg/ml Geneticin (Life Technologies).

Cells were harvested and centrifuged at 300 g for 5 minutes at 4 °C. The media was aspirated and the cells washed with chilled FACS buffer (DPBS, 2 mg/ml BSA, 0.05% Sodium Azide). For each of the four stable cell lines, 4 × 10^5^ cells were seeded into each well of a 96 well plate and centrifuged (300 × g, 5 mins, 4 °C). The wash buffer was aspirated and the cells incubated on ice for 1 hour in FACS buffer containing IVIg, γ1-hexameric-Fc or γ4-hexameric-Fc at concentrations ranging from 0.1 to 10 μg/ml.

The cells underwent two washes in 140 μl of chilled FACS buffer and incubated on ice for a further 30 minutes in FACS buffer containing an goat anti-human (H + L) F(ab′)2 R-Phycoerythrin conjugated antibody, diluted 1:200 (Jackson ImmunoResearch). This was followed by a further three washes in chilled FACS buffer, re-suspended in 100 μl buffer and run on BD FACSCanto. Data was analysed on FlowJo to calculate GeoMean of PE intensity for gated live cells.

### Analysis of hexameric-Fc internalization

Human monocyte-derived macrophages (HMDM) grown as previously described were transferred to a chambered coverglass (Nunc) and allowed to adhere. Cells were then incubated at 4 °C or 37 °C for 30 minutes with the indicated hexameric-Fc, followed by incubation at 4 °C with a goat anti-human AF647 secondary antibody (Life Technologies). Cells were then washed and fixed with 4% paraformaldehyde (PFA) in PBS for 10 minutes, before washing and imaging in PBS. Images were acquired using a Leica SP5 Confocal microscope with a 63x objective and appropriate excitation wavelengths and emission filters. Laser power and acquisition settings were kept constant between image acquisitions of different conditions. For uptake of transferrin, cells were incubated with 50μg/ml AF568 conjugated transferrin and AF488-conjugated hexameric-Fc for 30 minutes before cells were washed, fixed with 4% PFA, washed with PBS and visualised by confocal microscopy. Images shown are representative of cells from three independent experiments.

### Western Blotting

HMDM were plated in 6-well plates and incubated with hexameric-Fc for 24 hours. Cell lysates were prepared in RIPA buffer containing phosphatase and protease inhibitors (Life Technologies) and analysed by immuno-blotting using antibodies against CD16 (AF1597, R&D systems), CD32a (ab194937, Abcam), CD32b (ab54143, Abcam) and CD64 (ab140779, Abcam).

### Macrophage surface labelling

To assess surface labelling of Fcγ-receptors HMDM were plated in 96-well plates and incubated with hexameric-Fc or IVIg. Cells were then incubated at 4 °C with FITC-conjugated antibodies against CD16, CD32 or CD64 (BD Biosciences) or AF488-conjugated hexameric-Fc to assess surface binding. Cells were then fixed with 4% PFA for 10 minutes, washed with PBS, permeabilised with PBS containing saponin and labelled with DAPI. Cells were then washed and left in PBS for imaging. Imaging was performed on a Thermofisher Arrayscan using appropriate excitation and emission filtersets. Images were quantified using the Spotdetector algorithm which identifies cells by the DAPI label, and quantifies the FITC or AF488 labelling.

### Phagocytosis assays

Autologous B cells were prepared from stored non-adherent PBMCs by negative selection using MACS (B cell isolation kit II, Miltenyi Biotech) and labelled with CFSE (Molecular Probes). Differentiated macrophages and B cells were co-cultured at a 5:1 ratio in the presence of 0.1 µg/ml anti-CD20 mAb (Rituximab, Biogen Idec/Genentech) to induce antibody-dependent phagocytosis of the B cells. Hexameric-Fc or IVIg (Gamunex) were added at the indicated concentrations and the cells incubated at 37 °C 5% CO_2_ for 18 h before analysis of remaining B cell targets by flow cytometry. HMDM were distinguished by their auto-fluorescence/side-scatter properties and B cells by their CSFE labelling. % Inhibition of phagocytosis was calculated as ((Value-Background)/(Max-Background)) × 100% where Value = remaining B cells in presence of hexameric-Fc or control plus anti-CD20, Max = remaining B cells in absence of anti-CD20 and Background = remaining B cells in presence of anti-CD20 alone.

### Tetanus immune complex PBMC T cell assay

Tetanus toxoid immune complexes (TT-IC) were generated by incubation of tetanus toxoid (TT; Statens Serum Institut, Denmark) with HyperTet S/D hyper-immune IgG serum (Grifols, USA). ICs were prepared in 96-well round bottom cell culture plates as 2x (100 µl) solutions in complete RPMI-1640 (10% FCS) medium. Briefly, TT (2.0 µg/ml) was incubated with HyperTet (2.0 mg/ml) over-night at 4 °C. PBMCs were then labelled with CellTrace Violet (ThermoFisher) according to the manufacturer’s instructions. Labelled PBMCs (300,000/well) were subsequently added in the same volume to the 2x TT-IC solution to give rise to a final concentration of 1.0 µg/ml of TT complexed in 1.0 mg/ml of HyperTet. Other assay conditions included TT only (1.0 µg/ml), or TT-ICs with hexameric-Fc (50 µg/ml) or FcγR blocking antibodies (R&D Systems, AF1330 anti-CD16 and AF1597anti-CD16, 20 µg/ml per antibody). Cells were incubated for 6d at 37 °C. Subsequently, T cell division was assessed through flow cytometry (BD Fortessa) by determining the percentage of T cells having undergone CellTrace violet dye dilution compared to unchallenged control conditions gating on T cells using a CD3-APCH7 antibody (BD). To aid comparison between independent experiments, T cell division was normalised taking into account minimal and maximal responses within individual experiments.

### Murine ITP model and cytokine release

All mouse experiments were conducted under the regulation of A(SP)A 1986 on Project Licence 30/2899 granted by the Home Office UK. Work was approved by the internal AWERB review committee at UCB. Male Balb/c mice were obtained from Charles River, UK and were greater than 6 weeks of age at the start of studies. Platelet loss was induced by the intraperitoneal (i.p.) administration of 1 µg/mouse anti-CD41 antibody (MWReg30; EBioscience). Mice were dosed with 10 mg/kg hexameric-Fc intravenously (i.v.) at time points from 6 days to 1 hour prior to MWReg30. Blood samples were taken immediately prior to MWReg30 administration and again 24 hour post to measure baseline and final platelet numbers respectively. Platelet number was determined by flow cytometry. Briefly, whole blood was stained with CD45-PerCP.Cy5.5 and CD42d-PE (EBioscience). Platelets were judged to be the CD45-ve CD42d+ve population. Statistical analysis was carried out by one way ANOVA and Dunnetts multiple comparison test. Cytokine release in mice was measured using MSD kits (Meso Scale Discovery) for Il-6, Il-10, KC, IFNγ, TNFα and Il-1β.

### Murine investigation of hexameric-Fc FcRn binding mutants and IgG clearance

Human FcRn transgenic mice (Jackson Laboratories, US) were anesthetised and intravenously infused with 500 mg/kg of human IVIg (Human IgG 10% Gamunex-c, Talecris Biotherapeutics). 24 hours later animals were dosed with PBS or Fc Multimer mutants intravenously. Mice were bled at 0, 8, 24, 48, 72, 144 and 192 hours from antibody administration (20 µl). Serum levels of human IgG in the FcRn transgenic mouse were determined by ELISA or mass spectroscopy.

### Mouse PK

Male CD-1 mice were administered a 0.5 or 2 mg/kg IV bolus dose of ^125^I labelled (using iodogen method) PB334 (IgG1 starfish) or PB390 (IgG4 FALA). At selected time points up to three days plasma was collected from three mice per time point. Concentrations of protein bound radioactivity in plasma were determined by direct measurement for 1 minute in a gamma counter (Packard Cobra 11 Autogamma) with correction for background, counter efficiency and half-life of degradation of ^125^I. Values were corrected to calculate µg-equivalents of hexameric-Fc per mL of plasma. The analytical method employed measures a composite of ^125^I containing Fc-bearing forms in plasma; which may include intact hexameric-Fc, and smaller and larger Fc-containing moieties.

### Cynomolgus monkey PK

The cynomolgus monkey PK was conducted at Envigo CRS Limited (UK) in which an intravenous bolus injection of 2 mg/kg of hexameric IgG4eng F234L F296Y was given to three animals. The in-life experimental procedures to be undertaken during the course of this study were subject to the provisions of the United Kingdom Animals (Scientific Procedures) Act 1986 Amendment Regulations 2012 (the Act). The study complied with all applicable sections of the Act and the associated Codes of Practice for the Housing and Care of Animals used in Scientific Procedures and the Humane Killing of Animals under Schedule 1 to the Act. Blood samples for PK analysis were samples at pre-dose, and 5 minutes, 2, 6, 24, 48 and 72 hours after each dose.

Hexameric IgG4eng F234L F296Y was measured in cynomolgus monkey plasma samples by Liquid chromatography-electrospray ionisation tandem mass spectrometry (LC-ESI/MS/MS) assays using the signature peptide (NH2 - GLPSSIEK – COOH) with horse myoglobin as internal standard (NH2 – LFTGHPETLEK - COOH). The method had a limit of quantification of 1.00 μg/mL and a calibration curve ranging from 1.00 to 1000 μg/mL. Calibration curve and QC samples were prepared in cynomolgus monkey plasma and gave <15% bias. The analytical method employed measures a composite value of all human Fc forms in plasma; which includes intact hexameric-Fc, and smaller and larger Fc-containing moieties. Surface labelling of monocytes was carried out in whole blood samples at 0, 2, 6 and 72 hour time-points using an AF647-conjugated hexameric-Fc and a fluorescent anti-CD14 conjugate to identify monocytes before analysis by flow-cytometry.

### SPR experiments

His-tagged Fc gamma receptors were captured by an anti-his antibody on three flowcells of a CM5 Biacore sensor chip at three different levels and left to stabilize. γ4-hexameric-Fc was titrated from 1μM to 7.8 nM and γ1 hexameric-Fc from 2.5μM to 39 nM, over the three flowcells and allowed to dissociate. IgG4 was titrated between 50μM and 0.39μM over the flowcell and allowed to dissociate.

For FcRn based SPR analysis the FcRn protein was captured by an anti-B2M antibody and hexameric-Fc were titrated between 2.5μM and 39 nM at pH 6.

### Interaction Map analysis

The data from the SPR experiments were imported as tab separated text files into the evaluation software TraceDrawer (Ridgeview Instruments AB, Uppsala, Sweden) and further analyzed with the Interaction Map program (Ridgeview Diagnostics AB, Uppsala Sweden) to decipher the interaction heterogeneity. Interaction Map has been described in detail and validated previously^24^. In short, the method relies on the assumption that a heterogeneous interaction consists of a number of parallel one-to-one interactions with different kinetics and affinities, and that a measured binding curve is the sum of all individual one-to-one interaction curves. Interaction Map resolves the contributing interaction processes of a binding by applying a non-linear fitting algorithm to the curve, and presents each component as peaks in an on-off map (log(k_a_) vs log(k_d_)), with colors corresponding to their contribution to the measured curves (the parameter weight, %).

## Electronic supplementary material

Supplementary Information
