# Supplementary material for: Multivalent Fcγ-receptor engagement by a hexameric Fc-fusion protein triggers Fcγ-receptor internalisation and modulation of Fcγ-receptor functions
Source: Sci Rep. 2017 Dec 6;7:17049. doi: 10.1038/s41598-017-17255-8 (PMC5719016; doi:10.1038/s41598-017-17255-8)
Supplement: Supplementary file 1 — Supplementary Information [file 41598_2017_17255_MOESM1_ESM.pdf]

Multivalent Fcγ-receptor engagement by a hexameric Fc-fusion protein triggers Fcγ-receptor internalisation and modulation of Fcγ-receptor functions

[1]Qureshi, O.S., [1]Rowley, T.F., [1]Junker, F., [1]Peters, S.J., [1]Crilly, S., [1]Compson, J., [1]Eddleston, A., [2]Björkelund, H., [1]Greenslade, K., [1]Parkinson, M., [1]Davies, N., [1]Griffin, R., [1]Pither, T.L., [1]Cain, K., [1]Christodoulou, L., [1]Staelens, L., [1]Ward, E., [1]Tibbitts, J., [1]Kiessling, A., [1]Smith, B., [1]Brennan, F.R., [2]Malmqvist, M., [1]Fallah-Arani, F., [1]Humphreys, D.P.*

[1]UCB Pharma, 216 Bath Road, Slough SL1 3WE, UK

[2]Ridgeview Diagnostics AB, Uppsala Science Park, SE75183 Uppsala, Sweden

*To whom correspondence should be addressed: Dr. David Humphreys, UCB Pharma, 216 Bath Road, Slough SL1 3WE, UK; E-mail: david.humphreys@ucb.com

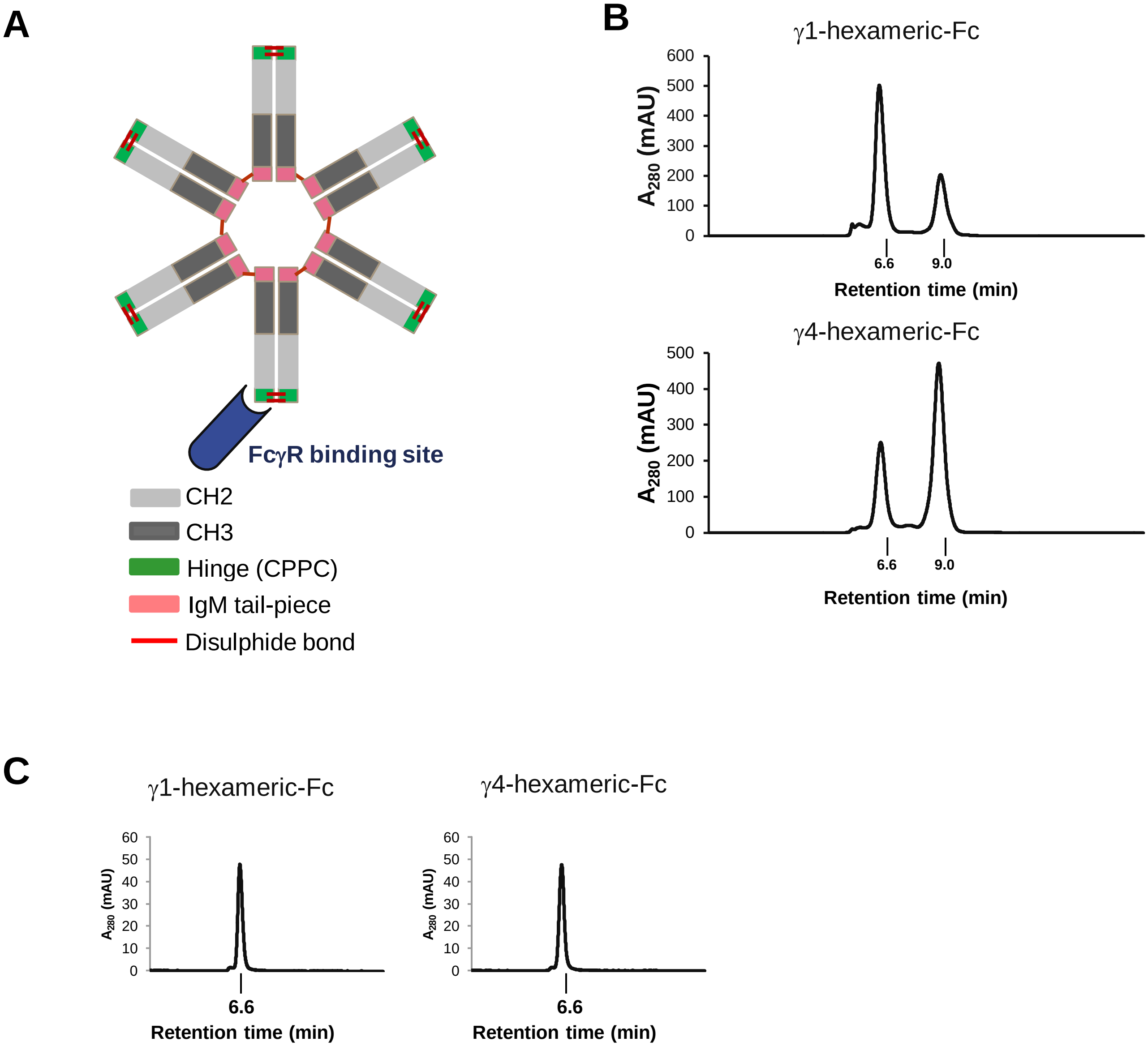

Supplementary Figure 1 – Generation of hexameric-Fc

A Schematic showing the design of hexameric-Fc. **B** Representative size exclusion chromatogram showing separation between hexamer and monomer post protein A purification. For γ1-hexameric-Fc, hexamer and monomer levels were observed around 70% and 30% respectively. For γ4-hexameric-Fc, typically 40% hexamer and 60% monomer was observed. **C** Representative size exclusion chromatograms of hexameric-Fc post gel filtration. All traces show pure hexamer fractions of at least 98%.

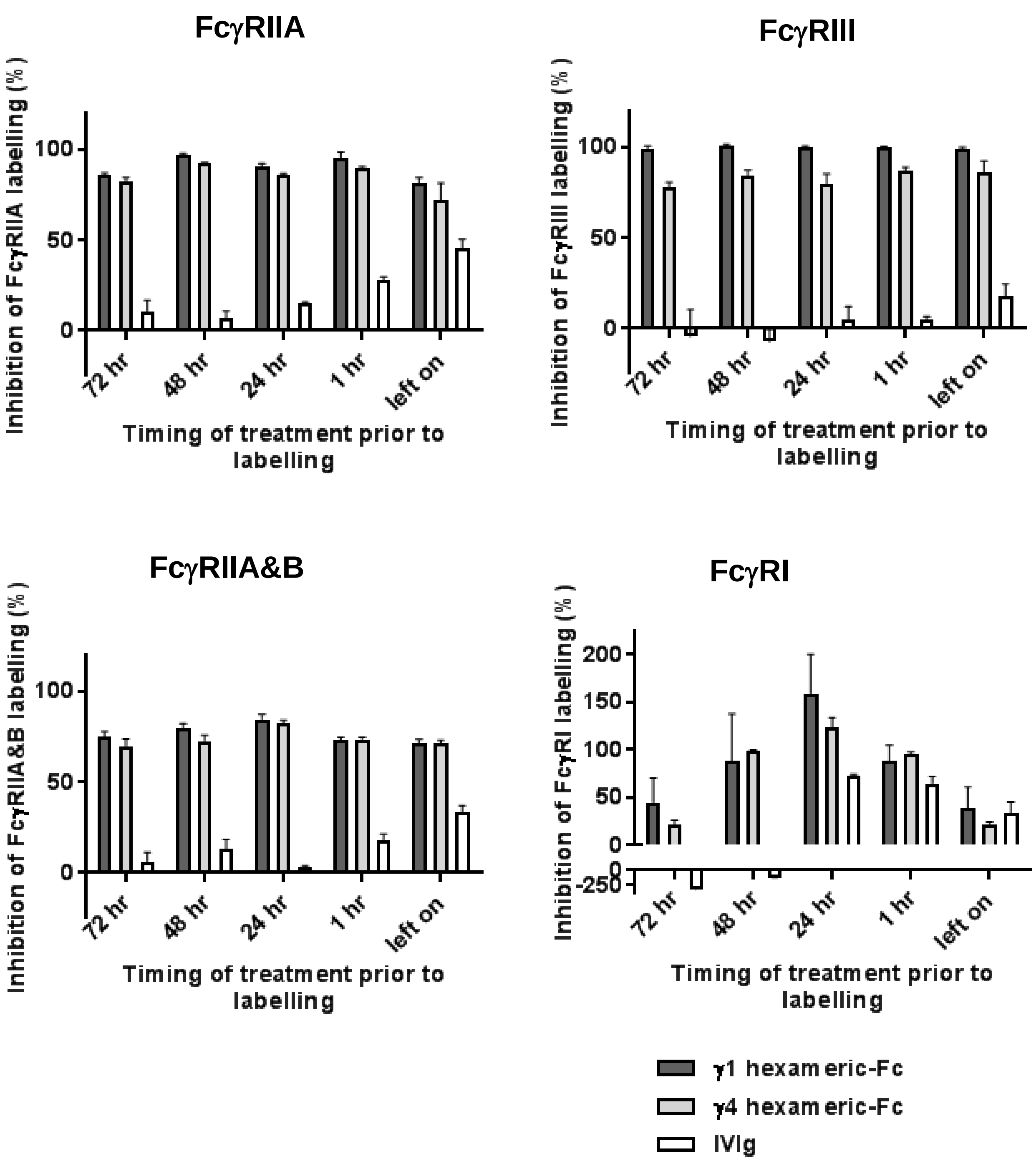

Supplementary Figure 2 – Prolonged disruption of FcγR surface labelling

Macrophages were incubated with hexameric-Fcs or IVIg at 75µg/ml for 1 hour. Cells were then washed and incubated for the indicated period. Cells were then labelled with fluorescently-conjugated antibodies against the indicated Fcγ-receptors. Data shows means from three donors ± SEM.

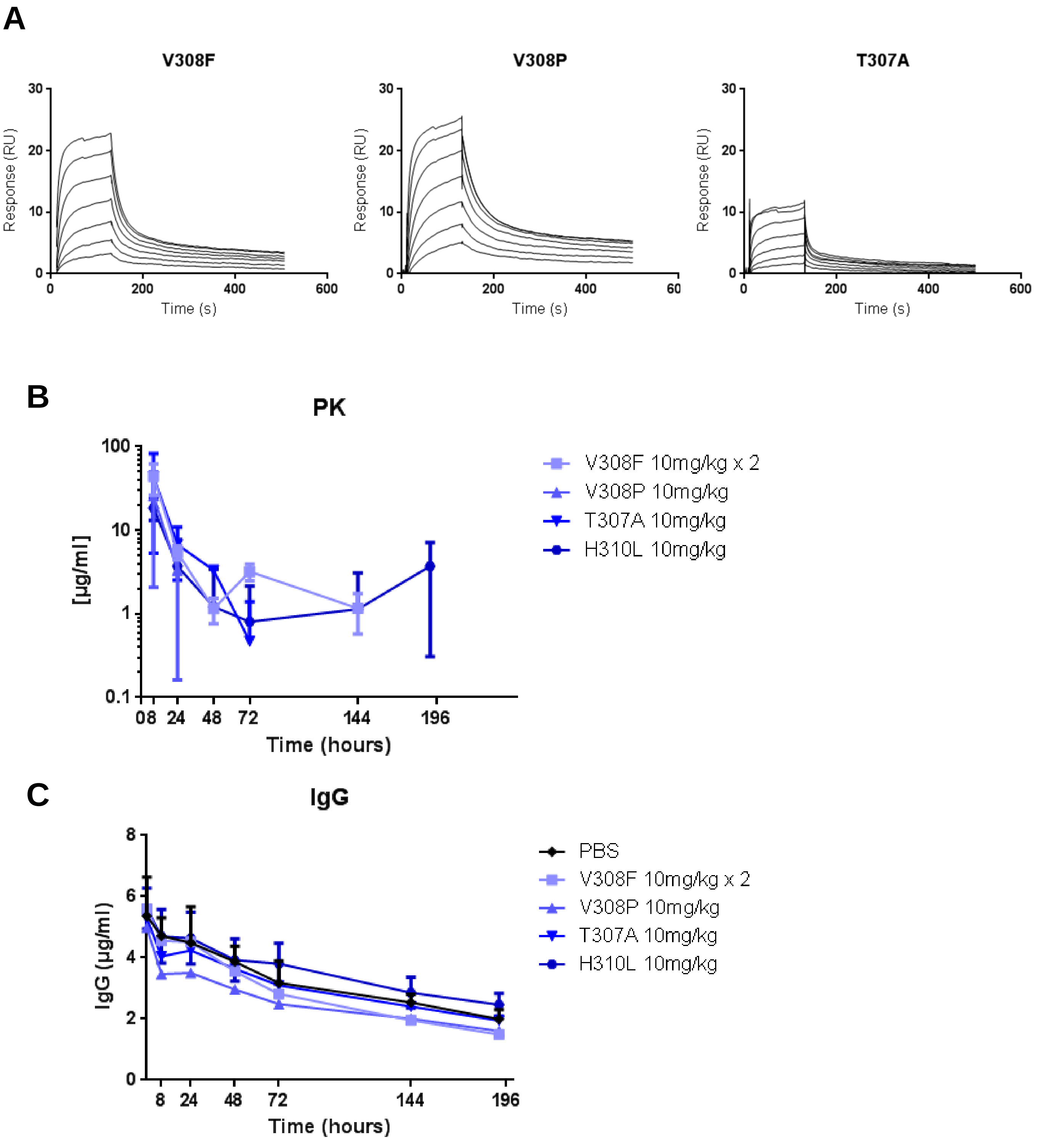

Supplementary Figure 3 – Effects of altering FcRn binding of hexameric-Fc

A Representative sensorgrams showing binding of hexameric-Fc variants binding to immobilised FcRn. Hexameric-Fc was titrated in a two-fold dilution series from 2.5µM to 39nM. **B** PK measurements in mice dosed with the indicated hexameric-Fcs. **C** Measurement of human IgG levels in mice dosed with indicated hexameric-Fcs.

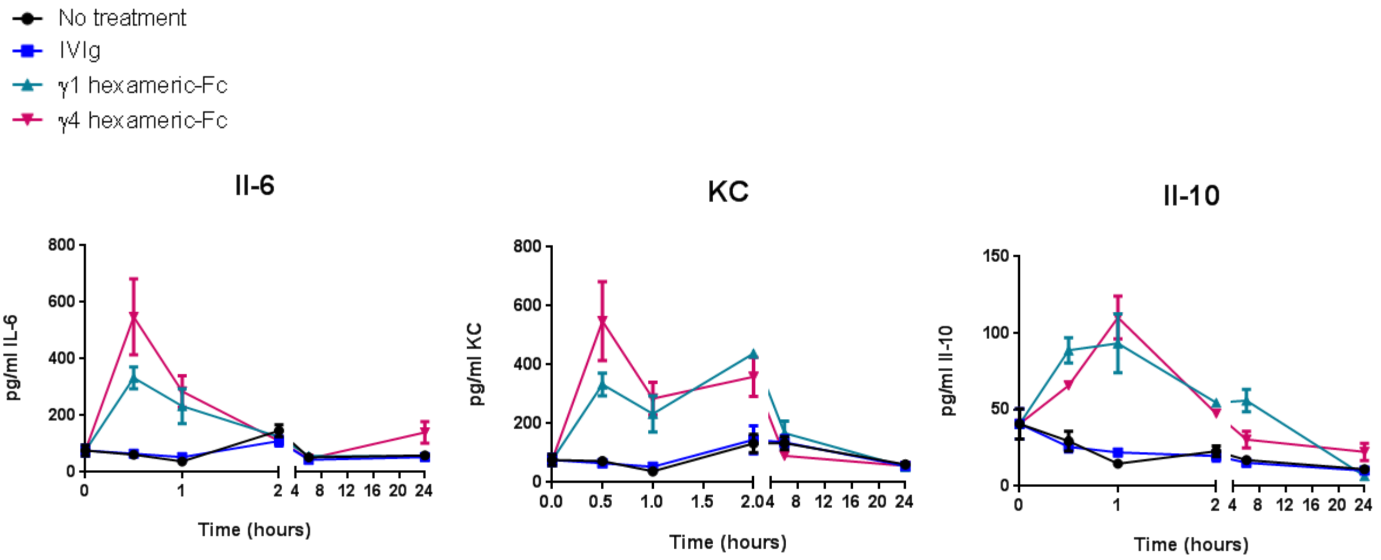

Supplementary Figure 4 – Effects of hexameric-Fc on cytokine release in mice

To assess the effect of hexameric-Fc on cytokine release, 10mg/kg hexameric-Fc was administered to mice IV. At the timepoints indicated, whole blood samples were taken and cytokine release measured by Meso Scale Discovery electrochemiluminescence assay. n = 5 mice per group.
